# Key Genes in the Melatonin Biosynthesis Pathway with Circadian Rhythm Are Associated with Various Abiotic Stresses

**DOI:** 10.3390/plants10010129

**Published:** 2021-01-09

**Authors:** Hye-Ryun Ahn, Yu-Jin Kim, You-Jin Lim, Shucheng Duan, Seok-Hyun Eom, Ki-Hong Jung

**Affiliations:** 1 Graduate School of Biotechnology & Crop Biotech Institute, Kyung Hee University, Yongin 17104, Korea; hrahn2018@khu.ac.kr; 2 Department of Life Science and Environmental Biochemistry, Pusan National University, Miryang 50463, Korea; yjkim2020@pusan.ac.kr; 3 Department of Horticultural Biotechnology, College of Life Sciences, Kyung Hee University, Yongin 17104, Korea; yujn0213@khu.ac.kr (Y.-J.L.); dsc97@khu.ac.kr (S.D.); se43@khu.ac.kr (S.-H.E.)

**Keywords:** abiotic stress, circadian rhythm, gene expression, melatonin biosynthesis, rice

## Abstract

Melatonin (*N*-acetyl-5-methoxytryptamine), a well-known animal hormone, is involved in several biological processes including circadian rhythm and the regulation of abiotic stress. A systematic understanding of the circadian regulation of melatonin biosynthesis-related genes has not been achieved in rice. In this study, key genes for all of the enzymes in the melatonin biosynthetic pathway that showed a peak of expression at night were identified by microarray data analysis and confirmed by qRT–PCR analysis. We further examined the expression patterns of the four genes under drought, salt, and cold stresses. The results showed that abiotic stresses, such as drought, salt, and cold, affected the expression patterns of melatonin biosynthetic genes. In addition, the circadian expression patterns of tryptophan decarboxylase (*TDC*), tryptamine 5-hydroxylase (*T5H*), and serotonin *N*-acetyltransferase (*SNAT*) genes in wild-type (WT) plants was damaged by the drought treatment under light and dark conditions. Conversely, *N*-acetylserotonin *O*-methyltransferase (*ASMT*) retained the circadian rhythm. The expression of *ASMT* was down-regulated by the rice *gigantea* (*OsGI*) mutation, suggesting the involvement of the melatonin biosynthetic pathway in the OsGI-mediated circadian regulation pathway. Taken together, our results provide clues to explain the relationship between circadian rhythms and abiotic stresses in the process of melatonin biosynthesis in rice.

## 1. Introduction

Melatonin, a well-known animal hormone, was first reported in plants in two articles [1,2]. Currently, melatonin has been identified in the roots, shoots, leaves, stems, fruits, and grains of various plant species [3]. Since its identification in plants, plant melatonin has been demonstrated to play a role in the 24 h cycle regulation [4,5,6], antioxidation [7,8,9,10], biotic and abiotic stress response [11,12,13,14], and plant growth and development [15,16,17,18,19].

**Figure 1:**
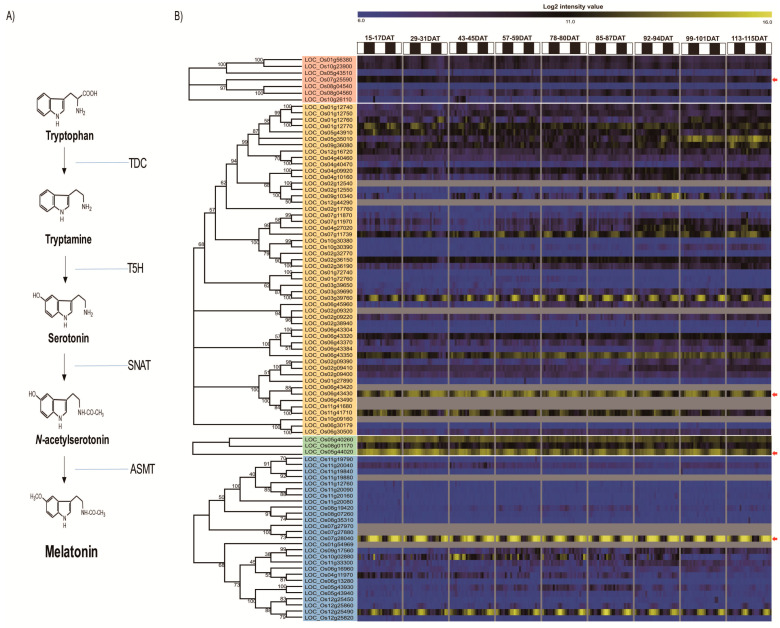
The standard pathway of melatonin biosynthesis from tryptophan in plants (**A**). Tryptophan decarboxylase (TDC); Tryptamine 5-hydroxylase (T5H); Serotonin N-acetyltransferase (SNAT); N-acetylserotonin O-methyltransferase (ASMT). Diurnal expression patterns of melatonin biosynthesis genes in rice, as assessed using Agilent 44k array data over the entire growth stage (**B**). Blue—low level of log2 intensity value; Yellow—high level of log2 intensity value.

The standard biosynthetic pathways of melatonin have now been established in plants, as shown in Figure 1A. In the melatonin biosynthetic pathway, tryptophan [20], a precursor of melatonin, is converted through four different enzymes [21,22,23]. The first enzyme is tryptophan decarboxylase (TDC), which converts tryptophan to tryptamine. The second enzyme, tryptamine 5-hydroxylase (T5H), catalyzes the conversion of tryptamine to serotonin [24]. Serotonin is then converted to *N*-acetylserotonin by a third enzyme, serotonin *N*-acetyltransferase (SNAT) catalysis, which is finally converted to *N*-acetyl-5-methoxytryptamine (melatonin) by the last enzyme, *N*-acetylserotonin *O*-methyltransferase (ASMT). TDC, T5H, SNAT, and ASMT are associated with melatonin biosynthesis in most plant species [25]. In rice, the functions of three TDC, one T5H, one SNAT, and three ASMT genes have been identified [26,27,28,29].

In recent years, many studies have focused on the function and regulation of melatonin in plants in their adaptation to abiotic stress. In particular, after treatment of several plant species with melatonin, a comparison with untreated plants was performed [19,22]. In mammals, melatonin has been clearly established as a modulator of the light and dark cycle [30,31,32]. Similarly, circadian rhythms exist in plants, and circadian oscillators can coordinate the stages of various biological processes. The concentration of melatonin in *Vitis vinifera*, a grape species, peaked at night under field conditions and was the lowest during the day with levels below 10 ng/g. Moreover, its levels were controlled by a 24 h cycle, suggesting that the decrease in melatonin during the light period is caused by melatonin consumption as an antioxidant response to solar radiation [33]. Two sweet cherry varieties, *Prunus avium* L. cv. Hongdeng and *Prunus avium* L. cv. Rainier, exhibit melatonin peaks twice (at 5h00 and 14h00) during the 24 h cycle. In addition, it was suggested that this is the role of the rate-limiting enzyme in the melatonin biosynthetic pathway of plants [34]. The leaves of *Malus zumi*, an apple species, also exhibit peaks at 5h30 and 14h30. It was also suggested that oxidative stress could induce melatonin biosynthesis [23].

The *TDC*, *T5H*, *SNAT*, and *ASMT* genes have been identified and functionally analyzed in many plants, including rice [28,35,36,37,38]. A genome-wide analysis of the *TDC* gene family was performed in *Solanum lycopersicum* [39]. As a result, it was determined that the tomato genome contains five TDC genes (*SlTrpDC1*–*SlTrpDC5*). Among them, *SlTDC1* and *SlTDC2* were specifically expressed in tomato fruits and leaves, respectively. The *ASMT* gene family was also analyzed at the whole-genome level [40]. The results revealed the existence of at least 14 members of this family, including three pseudogenes, and that some *SlASMTs* are induced by multiple pathogens. Moreover, it was suggested that these genes are involved in the responses of tomato plants to biotic stresses. Zhan et al. (2019) identified the *SNAT* and *ASMT* genes in 10 plants and performed phylogenetic analyses of these genes. In addition, an analysis of the expression profiles of wheat *TDC*, *T5H*, *SNAT*, and *ASMT* genes was performed under salt stress conditions [41]. It was found that many of these genes in wheat are up-regulated under salt conditions. In rice, a transcriptional profile analysis of melatonin synthesis and catabolic genes was performed, revealing a link between plant development and stress responses [42].

Rice is one of the most important crops worldwide, and genes encoding the enzymes that participate in the melatonin biosynthesis pathway are well conserved in plant and animal systems. Therefore, studies of endogenous melatonin in rice are very useful and important [42]. Melatonin is involved in various plant biological processes. In particular, circadian rhythms of melatonin appear to exist in plants, but further studies of the abiotic factors that affect endogenous melatonin levels should be conducted [22].

To date, rice genes encoding the stepwise sequence of four enzymes, TDC, T5H, SNAT, and ASMT, have been cloned and characterized. In this study, we performed a genome-wide analysis through the transcriptome data and identified 7 *TDC*, 52 *T5H*, 3 *SNAT*, and 27 *ASMT* genes in rice. To identify the rice genes in the melatonin biosynthetic pathway associated with the circadian rhythm, we performed a meta-expression profiling analysis of leaf samples collected every 2 h over a period of 48 h at the nine different developmental stages of rice. We identified a gene for each enzyme of the melatonin biosynthetic pathway that showed a peak of expression at night. In addition, we compared the expression of these genes between the *osphyb* mutant and the wild type. We expected that these results would provide clues to coordinate circadian regulation and stress response via melatonin biosynthesis in rice, as well as suggest a strategy for optimizing melatonin production in plant.

## 2. Materials and Methods

### 2.1. Identification of Melatonin Biosynthetic Genes in Rice

To identify all genes involved in the process of melatonin biosynthesis in rice (*Oryza sativa*), the previously characterized TDC, T5H, SNAT, and ASMT genes were searched for in GreenPhyl V4 (https://www.greenphyl.org/cgi-bin/index.cgi), which is a database for plant comparative genomics [26,27,28,29]. Moreover, we used the Simple Modular Architecture Research Tool (SMART, http://smart.embl-heidelberg.de/) and Pfam (http://pfam.xfam.org/) to confirm the existence of conserved domains among all the gene family members in each step of the melatonin biosynthesis pathway. Table S1 provides a list of the genes involved in the melatonin biosynthesis process in rice used in this study.

### 2.2. Multiple Sequence Alignment and Phylogenetic Analysis

For phylogenetic analysis of melatonin biosynthetic genes in rice, the Rice Genome Annotation Project database (RGAP, http://rice.plantbiology.msu.edu/) and phytozome (https://phytozome.jgi.doe.gov/pz/portal.html) were used to download the protein sequences. After sequence alignment using ClustalW [43], the phylogenetic analysis was performed using the Maximum Likelihood method in the MEGA7.0 program [44]. For the evaluation of the phylogenetic tree, we carried out a bootstrap analysis under 1000 replicates [45].

### 2.3. Analysis of the Diurnal Rhythm and Tissue-Specific Expression Using the Available Microarray and RNA-Seq Data

To identify rice genes in the melatonin biosynthetic pathway that exhibit a diurnal rhythm expression, raw microarray data (GSE36040) was downloaded from the Gene Expression Omnibus (GEO, http://www.ncbi.nlm.nih.gov/geo/), and we normalized the data as reported recently [46]. The transcriptome data used leaves sampled at 2 h intervals for 2 days during nine developmental stages [47]. To check the tissue-specific expression of rice genes in the melatonin biosynthetic pathway, raw RNA-Seq data for leaf blades, shoots, roots, young panicles, and seed [48] were downloaded from the Sequence Read Archive (SRA, https://www.ncbi.nlm.nih.gov/sra). After normalization as reported recently [49], the data were uploaded onto the Multi Experiment Viewer (MEV, http://www.tm4.org/mev.html). The data were visualized through a heatmap, and the heatmaps were further edited using Adobe Illustrator CS6.

### 2.4. Plant Materials and Stress Treatments

To investigate the functional association between the circadian rhythm and the rice *gigantea* (OsGI) gene, we germinated Dongjin (*Oryza sativa* L. ssp. *japonica* cv.) and OsGI mutant seeds in a Murashige and Skoog medium for 10 days at 28 °C/25 °C day/night temperatures, continuous light, and 78% humidity. We then transferred the seedlings to individual pots and placed them in a growth chamber (14 h light/10 h dark cycle, 28 °C (day)/25 °C (night), and 80% humidity; Younghwa Science, Daegu, Korea). At 30 days after germination, leaves were sampled at 2 h intervals for 24 h.

For the treatments of drought, salt, and cold stress [50], we placed 10-day-old Dongjin plants in water-removed conditions for drought stress treatment, in a 200 mM NaCl solution for salt stress, and in 4 °C ± 1 °C for cold stress using three time points, i.e., 0, 6, and 12 h. Subsequently, the leaves of four plants were collected and sampled as a biological replicate, and each treatment was repeated three times.

To examine the relationship between the melatonin biosynthetic pathway and the abiotic stress tolerance pathway, we used the rice phytochrome B (osphyb) mutant and wild-type (WT) plants. After 4 weeks of growth, we applied drought stress for 4 days and leaves were sampled during the day (10h00) and night (18h00) of the second and fourth days.

### 2.5. RNA Extraction and qRT–PCR Analysis

The leaf samples were frozen in liquid nitrogen immediately after sampling and ground with TissueLyser II (Qiagen, Hilden, Germany). Total RNA was extracted using RNAiso Plus according to the manufacturer’s protocol (TakaraBio, Kyoto, Japan). MMLV Reverse Transcriptase (Promega, WI, USA) and oligo (dT) primers were used for cDNA synthesis. We carried out a qRT–PCR analysis using a Rotor-Gene Q instrument system (Qiagen, Hiden, Germany), and the synthesized cDNAs were amplified using 2× Prime Q-Master mix with SYBR Green (GeNet Bio, Nonsan-Si, Korea).

For the normalization of the amplified transcripts, we used the rice ubiquitin 5 gene as an internal control of the qRT–PCR analysis (OsUbi5, LOC_Os01g22490) [51,52]. In addition, Late Elongated Hypocotyl (OsLHY, LOC_Os08g06110) was used as a positive control for the investigation of diurnal rhythms. The quality of samples under abiotic stress was monitored using OsbZIP23 (LOC_Os02g52780) [53] for drought and salt and OsNAC6 for cold (LOC_Os01g66120) [54] as positive controls (Figure S1). All primers used in these qRT–PCR analyses are summarized in Table S2.

### 2.6. Extraction of Melatonin

The extraction of melatonin was performed according to the previous method with some modification [55]. The fresh rice seedling samples were ground in liquid nitrogen using the TissueLyser (Qiagen, Hilden, Germany). The ground samples (0.1 g) were extracted with 1 mL of chloroform for 1 h at 23 °C in a shaking incubator (110 rpm). The extracts were centrifuged at 12,000 rpm for 10 min. The supernatants (1 mL) were filtered through a 0.45-µm hydrophilic PTFE membrane syringe filter (Futecs Co., Ltd., Daejeon, South Korea) and evaporated using a vacuum rotary evaporator (Eyela Co., Tokyo, Japan). The concentrated extracts were dissolved in 0.5 mL of 40% (*v*/*v*) aqueous methanol and subjected to HPLC.

### 2.7. Quantification of Melatonin Using HPLC Analysis

Analysis of melatonin was performed using a reversed-phase HPLC (Waters 2695 Alliance HPLC; Water Inc., Milford, MA, USA) with a polymethacrylate column (150 × 4.6 mm, 4 µm, Rspak DE-413, Shodex, Japan) according to the previous methods [56]. The flow rate was 0.2 mL/min by isocratic elution for 30 min, using water:acetonitrile (50:50). The injection volume was 10 µL and the column temperature was set at 30 °C. The eluents were monitored at 280 nm using a Waters 996 photodiode array detector (Waters, Inc.). Melatonin content in samples was quantified with a melatonin standard (Sigmaaldrich, St. Louis, MO, USA).

## 3. Results

### 3.1. Identification of Melatonin Biosynthetic Genes in Rice and Phylogenetic Analysis

To identify all possible genes involved in the process of melatonin biosynthesis in rice, the previously reported tryptophan decarboxylase (*TDC*; LOC_Os08g04540, LOC_Os07g25590, and LOC_Os08g04560), tryptamine 5-hydroxylase (*T5H*; LOC_Os12g16720), serotonin *N*-acetyltransferase (*SNAT*; LOC_Os05g40260), and *N*-acetylserotonin *O*-methyltransferase (*ASMT*; LOC_Os09g17560, LOC_Os10g02880, and LOC_Os10g02840) genes were used as queries in GreenPhyl V4 (Figure 1A). 

Consequently, we identified 7 *TDC*-, 112 *T5H*-, 3 *SNAT*-, and 30 *ASMT* family putative genes. Through domain analyses using the SMART and Pfam software, we identified the presence of the key domain of each enzyme in the melatonin biosynthetic pathway (Pyridoxal_deC in the TDC; Cytochrome p450 in the T5H; Acetyltransf_1,7,10 in the SNAT; and Dimerisation and Methyltransf_2 in the ASMT family) and selected 7 TDC, 52 out of 112 T5H, 3 SNAT, and 27 out of 30 ASMT genes for further analysis (Figure 1B). Subsequently, we generated a phylogenetic tree using MEGA7.0 and identified three subgroups for *TDC*, five for *T5H*, two for *SNAT*, and two for *ASMT*.

### 3.2. Diurnal and Circadian Transcriptional Profile of Melatonin Biosynthetic Genes throughout the Entire Growth Cycle of Rice

**Figure 2:**
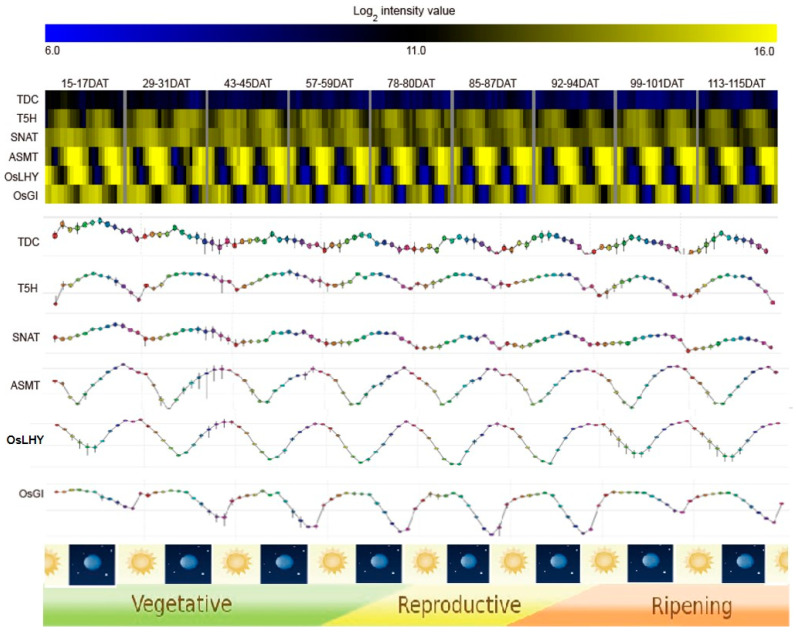
The meta-expression analysis of genes in each step of the rice melatonin biosynthetic pathway and confirmation of the expression patterns from RiceXPro. As standard marker genes for diurnal rhythm, the expression of *OsLHY* peaked during daytime and the expression of *OsGI* peaked at the start of the nighttime. *TDC*—LOC_Os07g25590; *T5H*—LOC_Os06g43430; *SNAT*—LOC_Os05g44020; *ASMT*—LOC_Os07g28040. DAT—days after transplanting.

To determine whether the melatonin biosynthesis-related genes are involved in diurnal regulation, we downloaded the publicly available Agilent 44k array data (GSE36040), which were produced by a diurnal regulation study using rice leaves collected from nine developmental stages from the NCBI gene expression omnibus (GEO, https://www.ncbi.nlm.nih.gov/geo/) [47]. The field leaf samples of each developmental stage of rice were collected every 2 h over 2 days. Through a meta-expression analysis using transcriptome data, we found a representative gene in each step of the pathway using the night peak expression patterns: *TDC* (LOC_Os07g25590), *T5H* (LOC_Os06g43430), *SNAT* (LOC_Os05g44020), and *ASMT* (LOC_Os07g28040). In addition, the diurnal expression patterns for the meta-expression data were evaluated using expression data of *OsLHY*, which is a marker gene that starts the expression at night and exhibits a peak at dawn, and *OsGI*, which is a marker gene showing a peak during the day. Subsequently, the diurnal patterns of these genes were further analyzed using RXP_0002 data from The Rice Expression Profile Database (RiceXPro; https://ricexpro.dna.affrc.go.jp/) website, which were also deposited as GSE36040 in GEO (Figure 2) [46]. As a result, we found that the expression patterns of four selected genes in the melatonin biosynthetic pathway exhibited a peak a little earlier than that of *OsLHY*, indicating that they are all activated during the night and are more probable candidate melatonin biosynthetic genes.

### 3.3. qRT–PCR Confirmation of the Diurnal Expression Patterns of Melatonin Biosynthetic Genes in Rice and Their Involvement in the OsGI-Mediated Circadian Regulation Pathway

**Figure 3:**
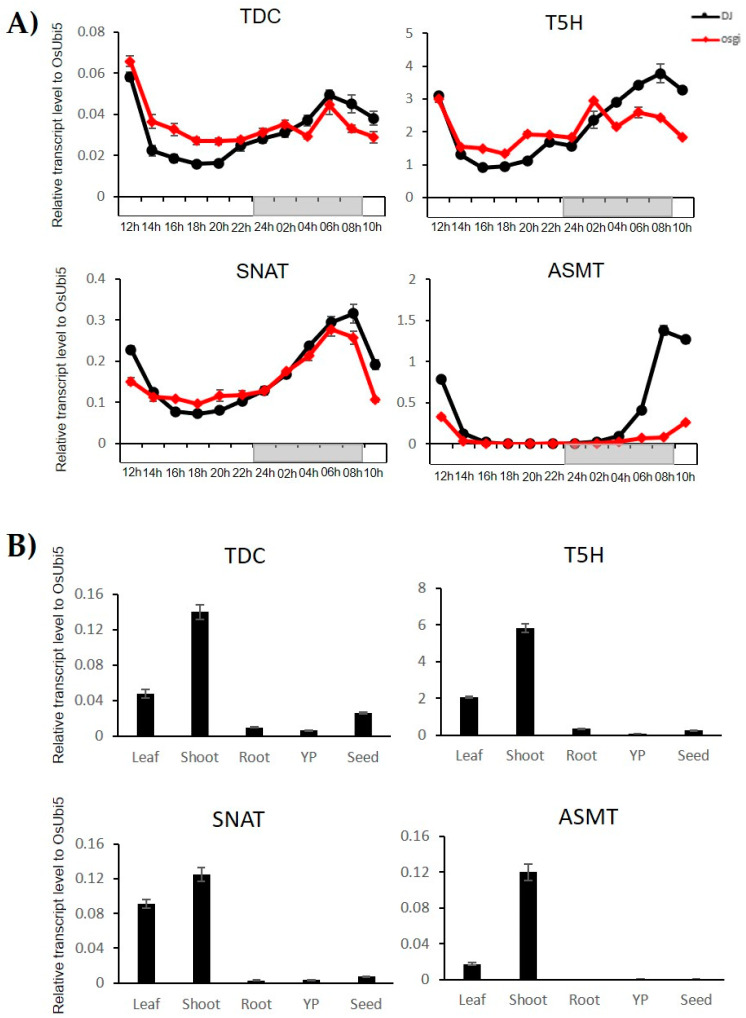
Expression profiles of four melatonin biosynthetic genes at 12 time points over a 24 h period in “Dongjin” rice (DJ) and the *OsGI* mutant (**A**), and in five tissues/organs (**B**). The continuous white and gray bars indicate day and night time, respectively. The anatomical samples used were five tissues/organs: leaf blade (LB), shoot, root, young panicle (YP), and seed. OsUbi5 was used as the internal control. Y-axis, relative expression level compared with OsUbi5.

To confirm the meta-analysis results of the anatomical expression profiles of the TDC, T5H, SNAT, and ASMT genes which show circadian rhythms (Figure S2), we performed qRT–PCR using shoots and roots of seedlings at 7 days after germination, leaves at 28 days, young panicles at 7 days before heading, and developing seeds at 6 days after fertilization. We confirmed their leaf-blade-preferred or shoot-preferred expression patterns (Figure 3A).

To confirm these expression patterns, we collected leaf samples from 4-week-old rice seedlings every 2 h over 24 h and carried out a qRT–PCR analysis. As expected, we confirmed the night peak expression patterns of the four selected genes (Figure 3B). Next, we compared the expression pattern data between wild-type rice and the *OsGI* rice mutant. We found that the expression patterns of the genes in first three steps of the pathway were not significantly different between wild-type and *OsGI* mutant plants. However, the *ASMT* (LOC_Os07g28040) was down-regulated by the *OsGI* mutation, indicating that the last step of the melatonin biosynthetic pathway might be regulated by the *OsGI* protein, which is a key component of the circadian clock in rice. These results indicate that the genes identified in this study in association with the melatonin biosynthetic pathway play roles in leaf and diurnal regulation.

### 3.4. Responses of the Melatonin Biosynthetic Genes to Drought, Salt, and Cold Stresses

**Figure 4:**
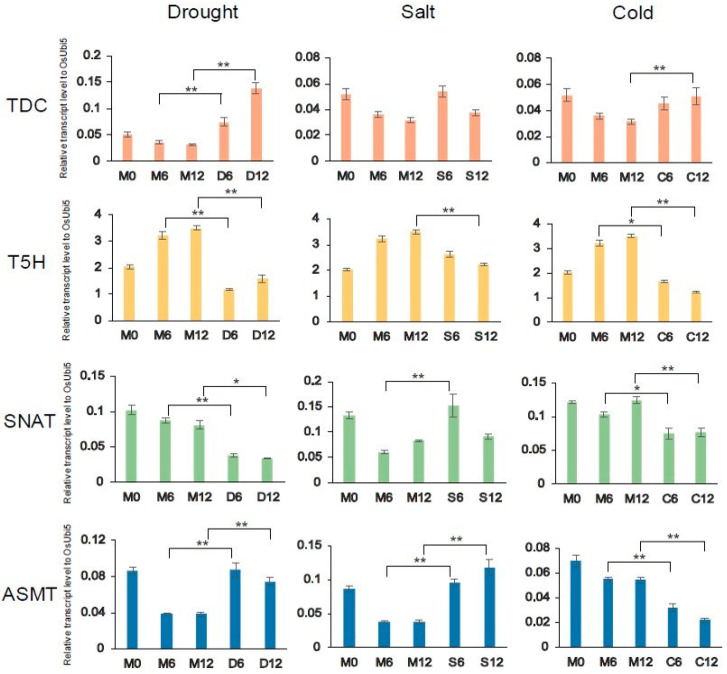
Expression profiles of four melatonin biosynthetic genes under drought, salt, and cold stress, as assessed by qRT–PCR analysis. *OsUbi5* was used as the internal control. M—mock; D—drought treatment; S—salt treatment; C—cold treatment. The numbers indicate the time points (h) after stress treatment. ** *p* < 0.01; * 0.01 < *p* < 0.05.

The involvement of melatonin in various stress responses is well known [57,58]. In particular, we were interested in the relationship between stress response and four selected genes in the melatonin biosynthetic pathway. To examine this relationship, we carried out an analysis of the expression patterns of the four genes under drought, salt, and cold stresses (Figure 4). First, drought stress triggered the expression of the TDC and ASMT genes, but repressed those of T5H and SNAT. In turn, salt stress up-regulated the expression of TDC, SNAT, and ASMT, but repressed that of T5H. Moreover, cold stress at 4 °C up-regulated slightly the expression of TDC, but repressed that of T5H, SNAT, and ASMT. Our results indicate that the melatonin biosynthetic pathway in rice can be modulated by various abiotic stresses. The detailed responses to three abiotic stresses might vary based on the expression patterns of four representative genes in the melatonin biosynthetic pathway of rice.

### 3.5. Response of Melatonin Biosynthetic Genes to Drought Stress Coupled with a Light–Dark Cycle

**Figure 5:**
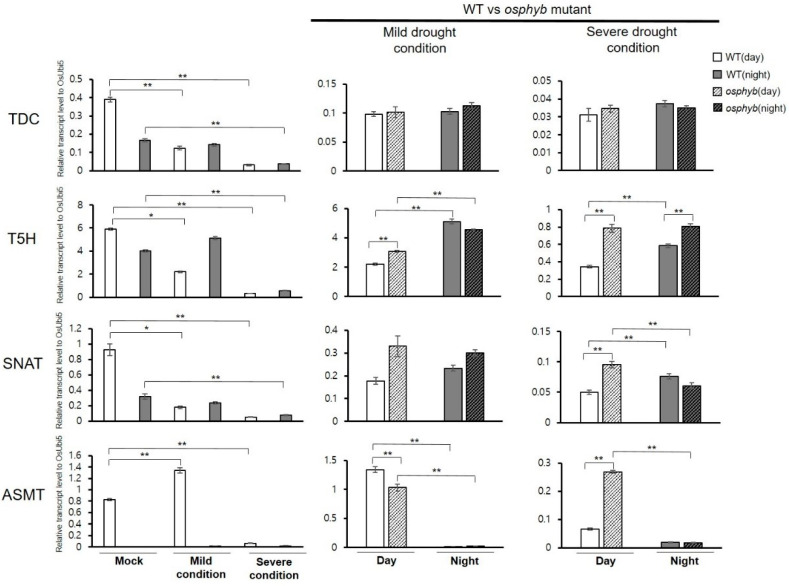
Expression profiles of four melatonin biosynthetic genes in response to drought stress coupled with a light–dark cycle in wild-type rice and the *osphyb* mutant. *OsUbi5* was used as the internal control. *osphyb*, knockout mutant of *OsPhyB*; WT—wild-type. ** *p* < 0.01; * 0.01 < *p* < 0.05.

In mammals, melatonin has been clearly established as a modulator of the light–dark cycle [30,31,32]. It is also known that melatonin in plants is regulated by a 24 h cycle and plays a role as a circadian oscillator to adjust the phase of a variety of biological processes, such as gene and metabolic regulation and protein stability [22]. Therefore, to determine if the genes selected among each of the melatonin biosynthetic enzyme families in rice exhibit a change in expression in response to abiotic stress coupled with light and dark conditions, drought treatment was performed under light and dark conditions. As a result, circadian expression patterns of *TDC*, *T5H*, and *SNAT* genes in wild-type (WT) plants were damaged by the drought treatment. Conversely, *ASMT* retained the circadian rhythm, and its expression was increased by 1.6-fold in mild drought conditions. Moreover, in severe drought conditions, the expression of all genes was repressed compared with the mock experiment (Figure 5).

Phytochrome B plays a role in photomorphogenesis [59]. It has been reported that *OsphyB* is negatively involved in drought resistance by controlling stomata density [60]. In addition, experiments using the *osphyb* mutant disrupted by T-DNA insertion and the segregating wild types suggested that the root development during drought stress is important for the tolerance response. By measuring the activity of ascorbate peroxidases (APXs) and catalases (CATs), which are known as important enzymes for H_2_O_2_ elimination, it was suggested that unlike the wild type in the same genetic background, *osphyb* mutants maintain the resistance against drought stress by effectively reactive oxygen species (ROS) scavenging in rice roots and finally overcome the unfavorable environment [61].

To examine the expression changes of melatonin biosynthetic genes in the drought-stress-resistant *osphyb* mutant, the expression of these genes was analyzed in wild-type and *osphyb* mutant plants after drought stress treatment. We found that the *osphyb* mutant retained the circadian expression pattern of *SNAT*; in contrast, under severe drought conditions, T5H, SNAT, and ASMT were up-regulated by ~2-fold in the *osphyb* mutant compared with wild-type plants (Figure 5). In addition, we found that melatonin level in *osphyb* mutant was higher than that in the wild type under normal conditions (Figure S3). This result may explain the drought-stress tolerance in the mutant. The detailed mechanism needs to be elucidated through further study. 

## 4. Discussion

Previous studies have shown that melatonin exhibits circadian rhythms in plants through the measurement of melatonin concentration in several plant species [22]. In addition, for the first time, a transcriptional profiling analysis of genes related to melatonin synthesis and catabolism showed circadian rhythms for *OsTDC3* and *Os2-ODD19* among 11 melatonin synthesis-related and catabolism-related genes [42]. Our analysis further identified the presence of circadian rhythms for genes in the whole pathway of rice melatonin biosynthesis (Figure 1B). The *TDC* (LOC_Os07g25590), *T5H* (LOC_Os06g43430), *SNAT* (LOC_Os05g44020), and *ASTM* (LOC_Os07g28040) genes identified in this study might be involved in the regulation of endogenous melatonin levels during the entire development period of rice (Figure 2). In *Chenopodium rubrum*, melatonin was detected at low or undetectable levels during the day period and showed a significant increase during the night period, a pattern that was similar to that observed in mammals [62].

Melatonin is involved in various stress responses [23,63,64,65,66,67,68]. In cadmium-treated rice leaves, endogenous melatonin was increased by sixfold compared with control plants, and *TDC*, *T5H*, and *HIOMT* were also up-regulated [68]. Four melatonin biosynthetic genes (*MdTDC1*, *MdAANAT2*, *MdT5H4*, and *MdASMT1*) were up-regulated after drought treatment in apples (*Malus* species) [69]. *TDC* expression was increased after drought, salt, and cold treatments, while *ASMT* expression was increased after drought and salt treatments (Figure 4). Our results indicate that the process of melatonin synthesis can be regulated by abiotic stresses in rice.

In soybean, drought stress reduced the expression of evening-specific factors (*TOC1*, *LUX*, *ELF4*, and *PRR*-like genes), leading to the destruction of the circadian system [70]. In *Arabidopsis*, heat stress has been reported to shorten the circadian period [71], whereas cold stress leads to the loss of the rhythm. In the 4 °C day and night cycle, most clock-related genes vibrate with decreasing amplitude and become irregular when moving with continuous light [72,73,74]. In Figure 5, the rhythm of expression of the TDC, T5H, and SNAT genes, but not of the *ASMT* gene, in the melatonin biosynthetic pathway during the drought stress treatment was destroyed, which provides evidence of the alteration of clock function according to abiotic stress conditions. In barley (*Hordeum vulgare* L.) and lupin, melatonin levels were increased significantly after osmotic stress [75]. In rice seedlings, melatonin concentration was increased at high temperature [76]. High expression of genes encoding melatonin synthetases (*TDC*, *T5H*, and *ASMT*) in rice grown in the presence of excess cadmium (Cd) is closely related to melatonin levels [68]. Increased production of melatonin in rice is associated with increased enzyme activity of SNAT and ASMT at high temperatures [76]. Our results also showed that abiotic stresses induce expression in the genes of melatonin biosynthesis pathway (Figure 5).

Phytochromes are a type of photoreceptor that primarily recognize and respond to red and far-red light to control various aspects of plant growth and development. In addition to the role of photomorphogenesis [59], cross talk between phytochrome-mediated light and drought-signaling pathways has been identified in various plants [77,78]. It has also been demonstrated that phyB or the R:FR ratio can decrease or increase the total leaf area depending on the growth conditions and plant species [79,80,81]. In Figure 5, the –2-fold increase in the expression of *T5H*, *SNAT*, and *ASMT* during daytime in the *osphyb* mutant compared with wild-type plants in severe drought conditions decreased the rate of recognition and response to light, resulting in a significant increase in melatonin according to the duration of the night period, while the decrease in the whole-leaf area in the *osphyb* mutant is recognized as a stressor.

In summary, in this study, we identified genes that exhibited a circadian rhythm and responded to abiotic stresses during the melatonin biosynthetic process in rice. Our results suggest that abiotic stress induces the expression of melatonin biosynthetic genes, which is mediated by night signals, suggesting their roles as biological agents. However, further studies are needed to obtain a deeper understanding of melatonin-mediated signaling as well as the basic molecular mechanisms underlying this process.

## Supplementary Materials

The following are available online at https://www.mdpi.com/2223-7747/10/1/129/s1, Table S1: List of the melatonin biosynthetic genes in rice used in this study, Table S2: List of the primers used for the qRT–PCR analysis, Figure S1: Quality check of the samples used for the analysis of diurnal rhythm or abiotic stress responses using marker genes, Figure S2: Meta-analysis of the RNA-Seq data of four melatonin biosynthetic genes, for the examination of anatomical expression, Figure S3: Melatonin levels of wild-type and *osphyb* mutant.

## References

[1] Dubbels R., Reiter R.J., Klenke E., Goebel A., Schnakenberg E., Ehlers C., Schiwara H.W., Schloot W. (1995). Melatonin in edible plants identified by radioimmunoassay and by high performance liquid chromatography-mass spectrometry. J. Pineal Res..

[2] Hattori A., Migitaka H., Iigo M., Itoh M., Yamamoto K., Ohtani-Kaneko R., Hara M., Suzuki T., Reiter R.J. (1995). Identification of melatonin in plants and its effects on plasma melatonin levels and binding to melatonin receptors in vertebrates. Biochem. Mol. Biol. Int..

[3] Debnath B., Islam W., Li M., Sun Y., Lu X., Mitra S., Hussain M., Liu S., Qiu D. (2019). Melatonin mediates enhancement of stress tolerance in plants. Int. J. Mol. Sci..

[4] Poeggeler B., Balzer I., Hardeland R., Lerchl A. (1991). Pineal hormone melatonin oscillates also in the dinoflagellate gonyaulax polyedra. Naturwissenschaften.

[5] Machácčková I., Krekule J. (2002). Sixty-five years of searching for the signals that trigger flowering. Russ. J. Plant Physiol..

[6] Kolář J., Johnson C.H., Macháčková I. (2003). Exogenously applied melatonin (N-acetyl-5-methoxytryptamine) affects flowering of the short-day plant Chenopodium rubrum. Physiol. Plantarum..

[7] Tan D.X., Manchester L.C., Reiter R.J., Qi W.B., Karbownik M., Calvo J.R. (2000). Significance of melatonin in antioxidative defense system: Reactions and products. Biol. Signals Recept..

[8] Van Tassel D.L., O’Neill S.D. (2001). Putative regulatory molecules in plants: Evaluating melatonin. J. Pineal Res..

[9] Cano A., Alcaraz O., Arnao M.B. (2003). Free radical-scavenging activity of indolic compounds in aqueous and ethanolic media. Anal. Bioanal. Chem..

[10] Tan D.X., Manchester L.C., Helton P., Reiter R.J. (2007). Phytoremediative capacity of plants enriched with melatonin. Plant Signal Behav..

[11] Zhang H.J., Zhang N., Yang R.C., Wang L., Sun Q.Q., Li D.B., Cao Y.Y., Weeda S., Zhao B., Ren S. (2014). Melatonin promotes seed germination under high salinity by regulating antioxidant systems, ABA and GA4 interaction in cucumber (*Cucumis sativus* L.). J. Pineal Res..

[12] Shi H., Tan D.X., Reiter R.J., Ye T., Yang F., Chan Z. (2015). Melatonin induces class A1 heat-shock factors (HSFA1s) and their possible involvement of thermotolerance in Arabidopsis. J. Pineal Res..

[13] Lee H.Y., Byeon Y., Back K. (2014). Melatonin as a signal molecule triggering defense responses against pathogen attack in Arabidopsis and tobacco. J. Pineal Res..

[14] Zhao H., Xu L., Su T., Jiang Y., Hu L., Ma F. (2015). Melatonin regulates carbohydrate metabolism and defenses against Pseudomonas syringae pv. tomato DC3000 infection in Arabidopsis thaliana. J. Pineal Res..

[15] Hernández-Ruiz J., Cano A., Arnao M.B. (2005). Melatonin acts as a growth-stimulating compound in some monocot species. J. Pineal Res..

[16] Hernández-Ruiz J., Cano A., Arnao M.B. (2004). Melatonin: A growth-stimulating compound present in lupin tissues. Planta.

[17] Arnao M.B., Hernández-Ruiz J. (2007). Melatonin promotes adventitious- and lateral root regeneration in etiolated hypocotyls of *Lupinus albus* L.. J. Pineal Res..

[18] Byeon Y., Back K. (2014). An increase in melatonin in transgenic rice causes pleiotropic phenotypes, including enhanced seedling growth, delayed flowering, and low grain yield. J. Pineal Res..

[19] Wei W., Li Q.T., Chu Y.N., Reiter R.J., Yu X.M., Zhu D.H., Zhang W.K., Ma B., Lin Q., Zhang J.S. (2015). Melatonin enhances plant growth and abiotic stress tolerance in soybean plants. J. Exp. Bot..

[20] Kang K., Kong K., Park S., Natsagdorj U., Kim Y.S., Back K. (2011). Molecular cloning of a plant N-acetylserotonin methyltransferase and its expression characteristics in rice. J. Pineal Res..

[21] Arnao M.B., Hernández-Ruiz J. (2014). Melatonin: Plant growth regulator and/or biostimulator during stress?. Trends Plant Sci..

[22] Arnao M.B., Hernández-Ruiz J. (2015). Functions of melatonin in plants: A review. J. Pineal Res..

[23] Zuo B., Zheng X., He P., Wang L., Lei Q., Feng C., Zhou J., Li Q., Han Z., Kong J. (2014). Overexpression of MzASMT improves melatonin production and enhances drought tolerance in transgenic Arabidopsis thaliana plants. J. Pineal Res..

[24] Posmyk M.M., Janas K.M. (2009). Melatonin in plants. Acta Physiol. Plant.

[25] Zhao H., Zhang K., Zhou X., Xi L., Wang Y., Xu H., Pan T., Zou Z. (2017). Melatonin alleviates chilling stress in cucumber seedlings by up-regulation of CsZat12 and modulation of polyamine and abscisic acid metabolism. Sci. Rep..

[26] Kang S., Kang K., Lee K., Back K. (2007). Characterization of rice tryptophan decarboxylases and their direct involvement in serotonin biosynthesis in transgenic rice. Planta.

[27] Fujiwara T., Maisonneuve S., Isshiki M., Mizutani M., Chen L., Wong H.L., Kawasaki T. (2010). Sekiguchi lesion gene encodes a cytochrome P450 monooxygenase that catalyzes conversion of tryptamine to serotonin in rice. J. Biol. Chem..

[28] Kang K., Lee K., Park S., Byeon Y., Back K. (2013). Molecular cloning of rice serotonin N-acetyltransferase, the penultimate gene in plant melatonin biosynthesis. J. Pineal Res..

[29] Park S., Byeon Y., Back K. (2013). Functional analyses of three ASMT gene family members in rice plants. J. Pineal Res..

[30] Reiter R.J. (1993). The melatonin rhythm: Both a clock and a calendar. Experientia.

[31] de la Iglesia H.O., Meyer J., Carpino A., Schwartz W.J. (2000). Antiphase oscillation of the left and right suprachiasmatic nuclei. Science.

[32] Reppert S.M., Weaver D.R. (2002). Coordination of circadian timing in mammals. Nature.

[33] Boccalandro H.E., Gonzalez C.V., Wunderlin D.A., Silva M.F. (2011). Melatonin levels, determined by LC-ESI-MS/MS, fluctuate during the day/night cycle in Vitis vinifera cv Malbec: Evidence of its antioxidant role in fruits. J. Pineal Res..

[34] Zhao Y., Tan D.X., Lei Q., Chen H., Wang L., Li Q., Gao Y., Kong J. (2013). Melatonin and its potential biological functions in the fruits of sweet cherry. J. Pineal Res..

[35] Byeon Y., Park S., Lee H.Y., Kim Y.S., Back K. (2014). Elevated production of melatonin in transgenic rice seeds expressing rice tryptophan decarboxylase. J. Pineal Res..

[36] Park S., Byeon Y., Back K. (2013). Transcriptional suppression of tryptamine 5-hydroxylase, a terminal serotonin biosynthetic gene, induces melatonin biosynthesis in rice (*Oryza sativa* L.). J. Pineal Res..

[37] Byeon Y., Lee H.Y., Lee K., Park S., Back K. (2014). Cellular localization and kinetics of the rice melatonin biosynthetic enzymes SNAT and ASMT. J. Pineal Res..

[38] Kang S., Kang K., Lee K., Back K. (2007). Characterization of tryptamine 5-hydroxylase and serotonin synthesis in rice plants. Plant Cell Rep..

[39] Pang X., Wei Y., Cheng Y., Pan L., Ye Q., Wang R., Ruan M., Zhou G., Yao Z., Li Z. (2018). The tryptophan decarboxylase in *Solanum lycopersicum*. Molecules.

[40] Liu W., Zhao D., Zheng C., Chen C., Peng X., Cheng Y., Wan H. (2017). Genomic analysis of the ASMT gene family in *Solanum lycopersicum*. Molecules.

[41] Zhan H., Nie X., Zhang T., Li S., Wang X., Du X., Tong W., Song W. (2019). Melatonin: A small molecule but important for salt stress tolerance in plants. Int. J. Mol. Sci..

[42] Wei Y., Zeng H., Hu W., Chen L., He C., Shi H. (2016). Comparative transcriptional profiling of melatonin synthesis and catabolic genes indicates the possible role of melatonin in developmental and stress responses in rice. Front. Plant Sci..

[43] Higgins D.G., Thompson J.D., Gibson T.J. (1996). Using CLUSTAL for multiple sequence alignments. Methods in Enzymology.

[44] Kumar S., Stecher G., Tamura K. (2016). MEGA7: Molecular evolutionary genetics analysis version 7.0 for bigger datasets. Mol. Biol. Evol..

[45] Kim E.J., Kim Y.J., Hong W.J., Lee C., Jeon J.S., Jung K.H. (2019). Genome-wide analysis of root hair preferred RBOH genes suggests that three RBOH genes are associated with auxin-mediated root hair development in rice. J. Plant Biol..

[46] Kim S.W., Lee S.K., Jeong H.J., An G., Jeon J.S., Jung K.H. (2017). Crosstalk between diurnal rhythm and water stress reveals an altered primary carbon fux into soluble sugars in drought-treated rice leaves. Sci. Rep..

[47] Sato Y., Takehisa H., Kamatsuki K., Minami H., Namiki N., Ikawa H., Ohyanagi H., Sugimoto K., Antonio B.A., Nagamura Y. (2013). RiceXPro Version 3.0: Expanding the informatics resource for rice transcriptome. Nucleic Acids Res..

[48] Sakai H., Mizuno H., Kawahara Y., Wakimoto H., Ikawa H., Kawahigashi H., Kanamori H., Matsumoto T., Itoh T., Gaut B.S. (2011). Retrogenes in rice (*Oryza sativa* L. ssp. japonica) exhibit correlated expression with their source genes. Genome Biol. Evol..

[49] Chandran A.K.N., Moon S., Yoo Y.H., Gho Y.S., Cao P., Sharma R., Sharma M.K., Ronald P.C., Jung K.H. (2019). A web-based tool for the prediction of rice transcription factor function. Database.

[50] Wu T., Zhang M., Zhang H., Huang K., Chen M., Chen C., Yang X., Li Z., Chen H., Ma Z. (2019). Identification and characterization of EDT1 conferring drought tolerance in rice. J. Plant Biol..

[51] Jain M., Nijhawan A., Tyagi A.K., Khurana J.P. (2006). Validation of housekeeping genes as internal control for studying gene expression in rice by quantitative real-time PCR. Biochem. Biophys. Res. Commun..

[52] Moon S., Chandran A.K.N., Kim Y.J., Gho Y., Hong W.J., An G., Lee C., Jung K.H. (2019). Rice RHC encoding a putative cellulase is essential for normal root hair elongation. J. Plant Biol..

[53] Xiang Y., Tang N., Du H., Ye H., Xiong L. (2008). Characterization of OsbZIP23 as a key player of the basic leucine zipper transcription factor family for conferring abscisic acid sensitivity and salinity and drought tolerance in rice. Plant Physiol..

[54] Ohnishi T., Sugahara S., Yamada T., Kikuchi K., Yoshiba Y., Hirano H.Y., Tsutsumi N. (2005). OsNAC6, a member of the NAC gene family, is induced by various stresses in rice. Genes Genet Syst..

[55] Lee K., Choi G.H., Back K. (2017). Cadmium-induced melatonin synthesis in rice requires light, hydrogen peroxide, and nitric oxide: Key regulatory roles for tryptophan decarboxylase and caffeic acid O-methyltransferase. J. Pineal Res..

[56] Arnao M.B., Hernández-Ruiz J. (2009). Assessment of different sample processing procedures applied to the determination of melatonin in plants. Phytochem. Anal..

[57] Sharma A., Zheng B. (2019). Melatonin mediated regulation of drought stress: Physiological and molecular aspects. Plants.

[58] Li J., Liu J., Zhu T., Zhao C., Li L., Chen M. (2019). The role of melatonin in salt stress responses. Int. J. Mol. Sci..

[59] Franklin K.A., Quail P.H. (2010). Phytochrome functions in Arabidopsis development. J. Exp. Bot..

[60] Liu J., Zhang F., Zhou J., Chen F., Wang B., Xie X. (2012). Phytochrome B control of total leaf area and stomatal density affects drought tolerance in rice. Plant Mol. Biol..

[61] Yoo Y.H., Nalini Chandran A.K., Park J.C., Gho Y.S., Lee S.W., An G., Jung K.H. (2017). OsPhyB-mediating novel regulatory pathway for drought tolerance in rice root identified by a global RNA-Seq transcriptome analysis of rice genes in response to water deficiencies. Front Plant Sci..

[62] Kolář J., Macháčková I., Eder J., Prinsen E., Van Dongen W., Van Onckelen H., Illnerová H. (1997). Melatonin: Occurrence and daily rhythm in *Chenopodium rubrum*. Phytochemistry.

[63] Shi H., Jiang C., Ye T., Tan D.X., Reiter R.J., Zhang H., Liu R., Chan Z. (2015). Comparative physiological, metabolomic, and transcriptomic analyses reveal mechanisms of improved abiotic stress resistance in bermudagrass [*Cynodon dactylon* (L). Pers.] by exogenous melatonin. J. Exp. Bot..

[64] Liang C., Zheng G., Li W., Wang Y., Hu B., Wang H., Wu H., Qian Y., Zhu X.G., Tan D.X. (2015). Melatonin delays leaf senescence and enhances salt stress tolerance in rice. J. Pineal Res..

[65] Turk H., Erdal S., Genisel M., Atici O., Demir Y., Yanmis D. (2014). The regulatory effect of melatonin on physiological, biochemical and molecular parameters in cold-stressed wheat seedlings. Plant Growth Regul..

[66] Tiryaki I., Keles H. (2012). Reversal of the inhibitory effect of light and high temperature on germination of Phacelia tanacetifolia seeds by melatonin. J. Pineal Res..

[67] Park S., Lee D.E., Jang H., Byeon Y., Kim Y.S., Back K. (2013). Melatonin-rich transgenic rice plants exhibit resistance to herbicide-induced oxidative stress. J. Pineal Res..

[68] Byeon Y., Lee H.Y., Hwang O.J., Lee H.J., Lee K., Back K. (2015). Coordinated regulation of melatonin synthesis and degradation genes in rice leaves in response to cadmium treatment. J. Pineal Res..

[69] Li C., Tan D.X., Liang D., Chang C., Jia D., Ma F. (2015). Melatonin mediates the regulation of ABA metabolism, free-radical scavenging, and stomatal behaviour in two Malus species under drought stress. J. Exp. Bot..

[70] Marcolino-Gomes J., Rodrigues F.A., Fuganti-Pagliarini R., Bendix C., Nakayama T.J., Celaya B., Molinari H.B., de Oliveira M.C., Harmon F.G., Nepomuceno A. (2014). Diurnal oscillations of soybean circadian clock and drought responsive genes. PLoS ONE.

[71] Kusakina J., Gould P.D., Hall A. (2014). A fast circadian clock at high temperatures is a conserved feature across Arabidopsis accessions and likely to be important for vegetative yield. Plant Cell Environ..

[72] Martino-Catt S., Ort D.R. (1992). Low temperature interrupts circadian regulation of transcriptional activity in chilling-sensitive plants. Proc. Natl. Acad. Sci. USA.

[73] Bieniawska Z., Espinoza C., Schlereth A., Sulpice R., Hincha D.K., Hannah M.A. (2008). Disruption of the arabidopsis circadian clock is responsible for extensive variation in the cold-responsive transcriptome. Plant Physiol..

[74] Espinoza C., Degenkolbe T., Caldana C., Zuther E., Leisse A., Willmitzer L., Hincha D.K., Hannah M.A. (2010). Interaction with diurnal and circadian regulation results in dynamic metabolic and transcriptional changes during cold acclimation in arabidopsis. PLoS ONE.

[75] Arnao M.B., Hernández-Ruiz J. (2009). Chemical stress by different agents affects the melatonin content of barley roots. J. Pineal Res..

[76] Byeon Y., Back K. (2014). Melatonin synthesis in rice seedlings in vivo is enhanced at high temperatures and under dark conditions due to increased serotonin N-acetyltransferase and N-acetylserotonin methyltransferase activities. J. Pineal Res..

[77] Ouedraogo M., Hubac C., Monard J.F. (1986). Effect of far red light on root growth and on xylem sap in cotton {*Gossypium hirsutum* L.). Plant Cell Physiol..

[78] Ouedraogo M., Hubac C. (1982). Effect of far red light on drought resistance of cotton. Plant Cell Physiol..

[79] Boccalandro H.E., Rugnone M.L., Moreno J.E., Ploschuk E.L., Serna L., Yanovsky M.J., Casal J.J. (2009). Phytochrome B enhances photosynthesis at the expense of water-use efficiency in arabidopsis. Plant Physiol..

[80] Lopez-Juez E., Buurmeijer W.F., Heerinca G.H., Kendrick R.E., Wesselius J.C. (1990). Response of light-grown wild-type and long hypocotyl mutant cucumber plants to endof-day far-red light. Photochem. Photobiol..

[81] Devlin P.F., Robson P.R., Patel S.R., Goosey L., Sharrock R.A., Whitelam G.C. (1999). Phytochrome D Acts in the shade-avoidance syndrome in arabidopsis by controlling elongation growth and flowering time. Plant Physiol..

