# Supplementary figures and images for: Key Genes in the Melatonin Biosynthesis Pathway with Circadian Rhythm Are Associated with Various Abiotic Stresses
Source: Plants (Basel). 2021 Jan 9;10(1):129. doi: 10.3390/plants10010129 (PMC7827461; doi:10.3390/plants10010129)

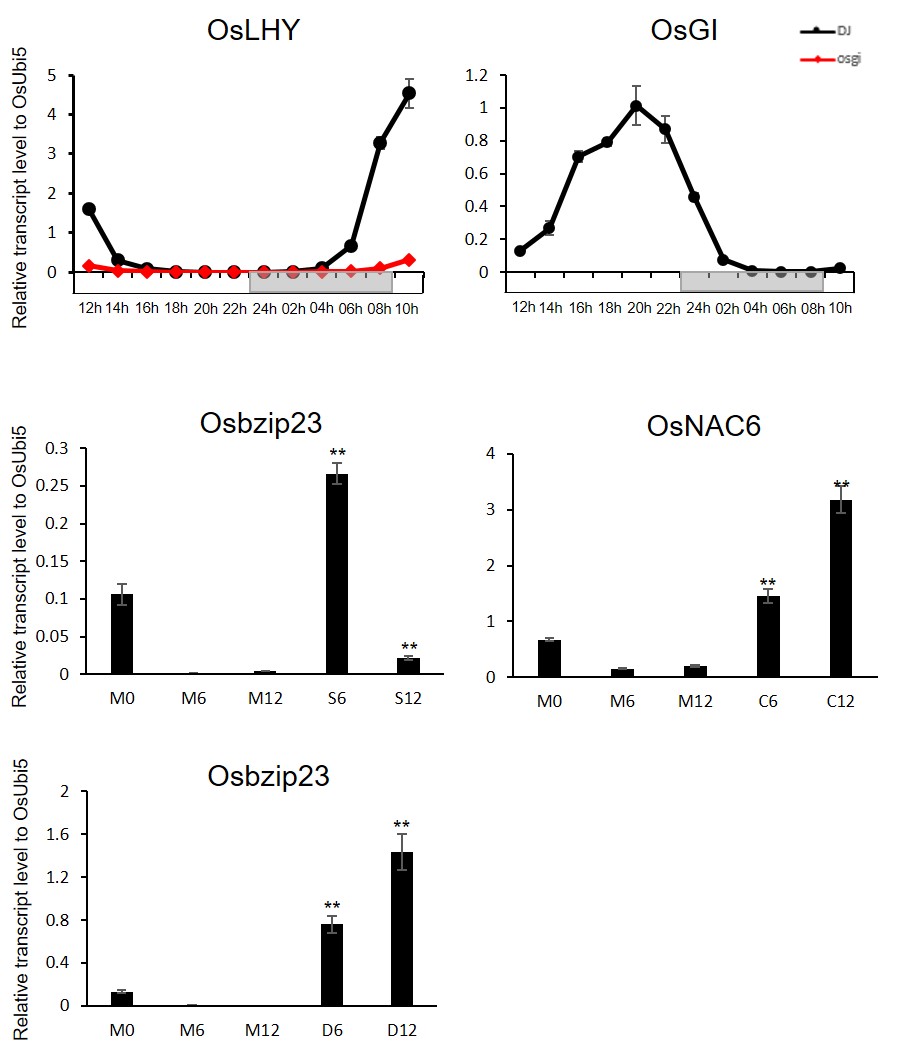

Supplement: Supplementary file 1 [file plants-10-00129-s001.zip › Figure S1.jpg]

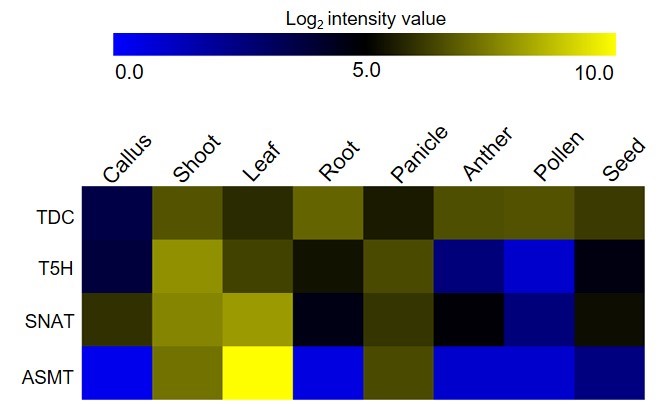

Supplement: Supplementary file 1 [file plants-10-00129-s001.zip › Figure S2.jpg]

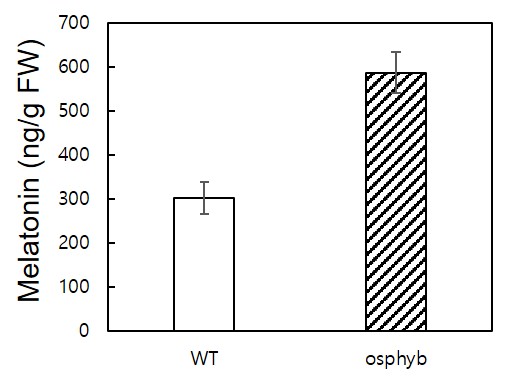

Supplement: Supplementary file 1 [file plants-10-00129-s001.zip › Figure S3.jpg]
